# SIMPATICO—Streamlined Isolation of the N-Terminome *via* Phosphonate Tagging and Coordination-Based Depletion

**DOI:** 10.1016/j.mcpro.2026.101660

**Published:** 2026-09-10

**Authors:** Piero Giansanti, Adnan Fojnica, Andreas Pichlmair

**Affiliations:** 1 Bavarian Center for Biomolecular Mass Spectrometry at the University Hospital rechts der Isar BayBioMS@MRI, School of Medicine and Health, Technical University of Munich, Munich, Germany; 2 TranslaTUM, Center for Translational Cancer Research, Technical University of Munich, Munich, Germany; 3 Institute of Virology, School of Medicine and Health, Technical University of Munich, Munich, Germany; 4 Institute of Virology, Munich, Germany; 5 German Centre for Infection Research (DZIF), Munich, Germany

**Keywords:** N-terminomics, NHS chemistry, Phosphonate, TMT, Caspase, Proteolysis, Label-free quantification

## Abstract

Protein processing is critical for regulating cellular functions. Therefore, there is considerable interest in developing effective proteome-wide strategies to identify protease cleavage products to enhance our understanding of proteolytic pathways and their perturbation in diseases. Here, we present a streamlined N-termini proteome analysis leveraging N-Hydroxysuccinimide (NHS) ester chemistry. At the protein level, N-terminal amines (naturally occurring protein N-termini, lysines, or protease-generated N-termini) are blocked directly in the cell lysate, and tryptic digestion is performed straight after, without the need for any buffer exchange. The internal tryptic peptides are tagged with a phosphonate moiety and subsequently depleted *via* metal-based coordination, keeping exclusively N-blocked terminal peptides for analysis by mass spectrometry. We demonstrate the applicability of this approach by monitoring proteolytic events induced by intrinsic apoptotic pathway. Our approach identified more than 690 aspartyl-cleaved proteins in response to Staurosporine-induced apoptosis, including many previously unknown substrates and cleavage sites. The presented approach is, therefore, a straightforward and robust method for mass spectrometry-based identification of caspase-generated cleavage products. Although demonstrated here in the context of caspase-mediated proteolysis, the underlying workflow is not restricted to this protease class and should be broadly applicable to the study of other proteolytic events.

## Highlights

• SIMPATICO workflow simplifies N-terminomics sample preparation.
• Direct “in-lysate” sample processing, eliminating buffer exchange steps.
• Phosphonate tagging enables efficient depletion of internal peptides.
• Identified >690 aspartyl-cleaved proteins during Staurosporine-induced apoptosis.
• Discovered numerous previously unknown caspase substrates and cut sites.

## Graphical Abstract

**Figure:**
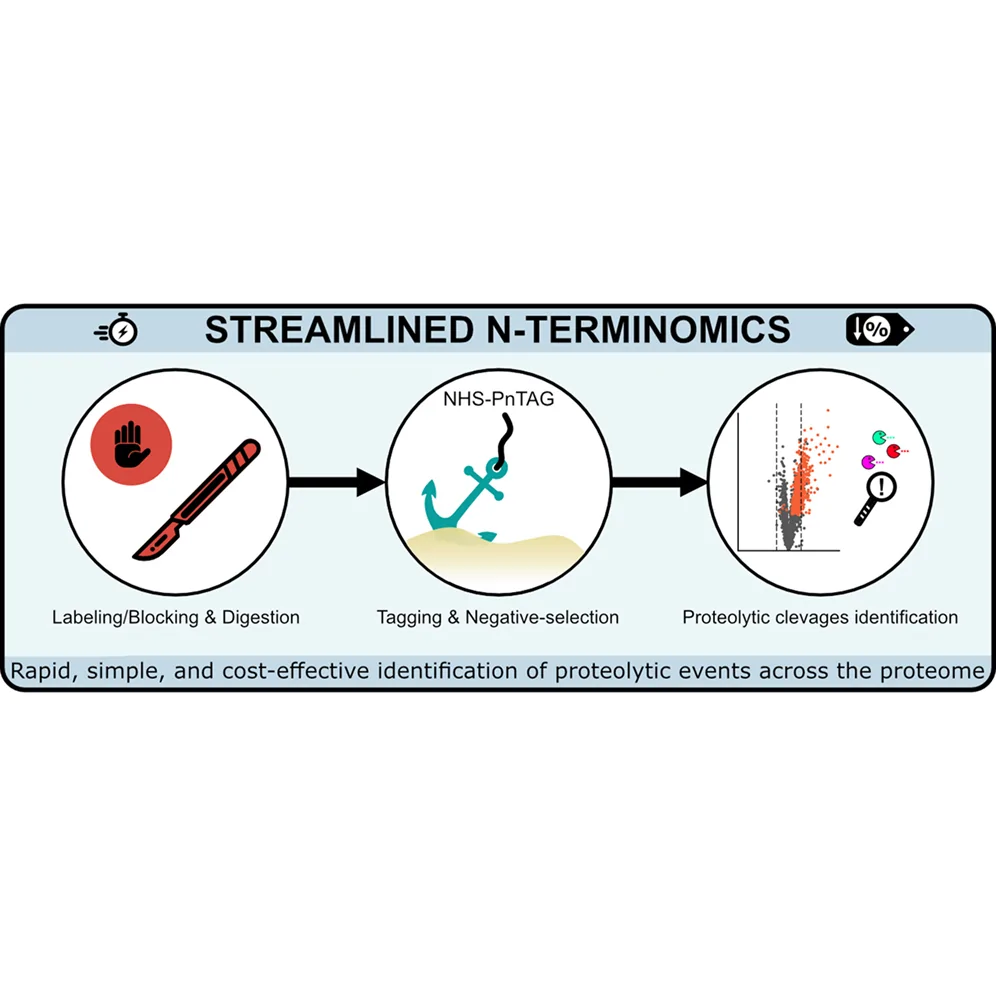

## In Brief

SIMPATICO is a streamlined and cost-effective N-terminomics workflow that enables direct, in-lysate sample processing without buffer exchange and supports both TMT-based multiplexing and label-free quantification. By combining NHS-based N-terminal blocking with phosphonate-mediated depletion of internal peptides, it efficiently enriches protein N-termini for mass spectrometry analysis. Applied to staurosporine-induced apoptosis, SIMPATICO identified more than 690 caspase-cleaved proteins, including numerous previously unknown substrates and cleavage sites, highlighting its power for comprehensive mapping of proteolytic events.

Proteolysis by endogenous proteases is a fundamental regulatory mechanism that impacts nearly every aspect of cellular function, from signal transduction to differentiation, protein turnover, and immune responses (1, 2, 3). Many physiological and pathological processes, including cancer progression, inflammation, and viral infections, are also driven by protease activity (4, 5, 6). Understanding proteolytic events is, therefore, crucial for deciphering biological processes and disease mechanisms, and it also facilitates the development of targeted therapeutics and biomarkers. In fact, by characterizing protease–substrate interactions, novel regulatory mechanisms can be uncovered, and potential drug targets for therapeutic intervention can be identified (7, 8, 9).

Despite its importance, systematically mapping protease substrates and cleavage sites remains a challenging task due to the transitory and dynamic nature of proteolytic events (10). Mass spectrometry-based proteomics has played a critical role in characterizing protein cleavage products at a global scale (11, 12, 13, 14, 15). Cravatt and coworkers pioneered a proteomic approach that enabled proteome-wide identification of protease substrates (16), later elegantly extended to a quantitative level using SILAC (17, 18). These methods enable the identification of proteolytic events by detecting shifts in protein migration on SDS-PAGE, providing insights into protease activity and substrate specificity. However, they are effective when proteolytic events cause a substantial shift in molecular weight, and are generally limited in precisely mapping cleavage sites, in detecting low-abundance cleavage products, and in scaling for high-throughput analysis due to limited multiplexing and extensive MS analysis required. Therefore, positional proteomics (19), and in particular N-terminomics, has emerged as a highly effective alternative for the in-depth and high-throughput characterization of proteolytic cleavage events (20), which addresses some of the limitations of the aforementioned methods. The rationale behind N-terminomics is that proteolysis generates new N-termini, which can be selectively exploited to determine precise cleavage sites. Techniques such as TAILS (21), and HUNTER (22) leveraged this principle to scavenge for protease-generated N-termini by taking advantage of the unique chemical structure and reactivity of the primary amines, enabling the direct identification of cleavage sites with high sensitivity.

These approaches follow a general workflow consisting of five main steps: (*i*) protein extraction and denaturation to improve the accessibility of amino groups, (*ii*) blocking/labeling of both protein N-termini (canonical or protease-generated) and lysine side chains, leveraging their readily reactive α- and ε-amines, (*iii*) proteome digestion, (*iv*) chemical tagging of internal peptides generated upon digestion containing newly exposed α-amines, and (*v*) selective depletion of internal peptides making use of the introduced tag.

Despite their efficacy, these methods have several drawbacks that limit their applicability for high-throughput analysis: the experimental workflow is lengthy and tedious, requiring two rounds of proteome cleanup, either through standard organic solvent-induced precipitation alone or in combination with capture onto hydroxylated magnetic beads (23, 24) to remove interfering compounds from the protein lysate, and before digestion. This not only increases time and cost but also introduces variability due to potential sample loss, especially since small cleavage products do not precipitate as efficiently as larger proteins (22, 25, 26). Additionally, despite cleanup, blocking/labeling reagents are typically used in high molar excess, with protein-to-reagent ratios ranging from 1:4 to 1:16, for TMT10 or TMTpro (23, 27, 28). Finally, the negative selection of the N-terminome often relies on a Schiff’s base reaction using cyanoborohydride, a highly toxic reagent that also produces hazardous byproducts, necessitating careful handling.

In view of these challenges, we have developed SIMPATICO, a streamlined isolation of the N-terminome *via* phosphonate tagging and coordination-based depletion. The method is built on the PTAG approach (29), but it radically minimizes processing steps and eliminates the need for toxic reagents. It relies exclusively on the well-established and widely used NHS esters chemistry, employing inexpensive reagents and standard laboratory equipment, making it easily accessible for any proteomics laboratory.

We demonstrate that SIMPATICO is a straightforward and efficient workflow, enabling the identification of more than 900 cleavage events in response to Staurosporine-induced apoptosis, and it can seamlessly integrate with multiplexing and label-free quantitative analysis.

## Experimental Procedures

### Cell Culture, Stimulation, and Lysate Preparation

HeLa S3 cells were cultured in Dulbecco's modified Eagle medium (Sigma-Aldrich), while Jurkat cells were maintained in Roswell Park Memorial Institute 1640 medium (Sigma-Aldrich), both supplemented with 10% fetal calf serum (GE Healthcare) and antibiotics (100 U/ml penicillin, 100 μg/ml streptomycin, Sigma-Aldrich). Cells were incubated at 37 °C in a humidified atmosphere (95% humidity, 5% CO_2_) and routinely tested for *Mycoplasma* contamination.

Jurkat cells were seeded at 1 × 10^6^ cells/ml before apoptosis induction. Staurosporine (STS, 1 μM, MedChemExpress) was added, and the cells were incubated for 6 h at 37 °C. HeLa and STS-treated Jurkat cells were then harvested, washed three times with phosphate-buffered saline (Sigma-Aldrich), snap-frozen in liquid nitrogen, and stored at −80 °C until further use.

Pellets were thawed on ice, and cells were lysed in lysis buffer containing 2 or 4% SDC (w/v, Sigma) and 200 mM HEPPS (Sigma), pH 8.5, by heating them for 5 min at 95 °C under shaking at 1500 rpm. The lysates were then sonicated with a Bioruptor Pico (Diagenode) for 10 ON/OFF cycles of 30 s. Protein concentration was estimated using a BCA assay (Thermo Fisher). To reduce and alkylate proteins, samples were incubated for 10 min at 45 °C with 10 mM tris(2-carboxyethyl) phosphine (Thermo Scientific) and 40 mM 2-chloroacetamide (Sigma).

### Primary Amines Blocking

The protocol for NHS ester-mediated blocking of primary amines was adapted from Zecha *et al*. (30).

For initial method optimization, individual channels of TMT10plex reagent (Thermo Scientific) were brought to RT and dissolved in anhydrous ACN under Argon gas. Varying amounts of reagents were mixed with varying amounts of cell lysate, maintaining a final ACN concentration of 20% (v/v), and incubated for 1 h at 20 °C in a thermoshaker with shaking at 500 rpm. Respective volumes and concentrations are specified in the results section. Afterward, ammonium bicarbonate (ABC, Sigma) was added to quench the reaction to achieve a final concentration of 50 mM, obtaining a final concentration of 0.24% SDC (w/v) and 6% ACN (v/v) for the lysate containing 1% SDC (v/v), and of 0.4% SDC (w/v) and 5% ACN (v/v) for the lysate containing 2% SDC (v/v).

For the multiplexing experiments, 0.6 mg of TMT10plex reagents (Thermo Scientific) were dissolved in anhydrous ACN and added to the samples (200 μg in 125 μl, 1% SDC), ensuring a final ACN concentration of 20% by volume. The blocking reaction proceeded as described above. ABC was added to quench the reaction to achieve a final concentration of 50 mM, obtaining a final concentration of 0.24% SDC (w/v) and 6% ACN (v/v). Quenching was performed for 30 min at 25 °C and 500 rpm, after which the labeled samples were combined.

For the label-free experiments, 2,5-dioxopyrrolidin-1-yl 2-methyl-5,8,11-trioxa-2-azatetradecan-14-oate (DMA-PEG3-NHS) was custom-synthesized by JenKem Technology upon request and used as blocking reagent for primary amine. Each sample received 0.67 mg of reagent dissolved in dry ACN while maintaining a final ACN concentration of 20% (v/v). Blocking and quenching were performed as described previously.

### Protein Digestion and Cleanup

Digestion was performed by adding trypsin (Thermo Scientific) at a 1:50 enzyme-to-substrate ratio followed by incubation overnight at 37 °C with continuous shaking at 700 rpm. Afterward, water was added to reduce ACN concentration to 3.8% (v/v), and digestion was stopped by acidification with neat formic acid (FA, Carlo Erba) to a final concentration of 5% (v/v). SDC and other insoluble matter were removed *via* centrifugation at 20.000*g* and 4 °C for 10 min.

Solid-phase extraction cartridges were used for desalting. For experiments involving TMT-multiplexing, a 200 mg, 3cc C18 SepPak column (Waters) was used, whereas for label-free experiments, a 50 mg, 1cc tC18 SepPak column (Waters) was utilized for each sample. The C18 sorbent material was conditioned by washing with pure ACN, followed by 80% ACN/0.1% FA (v/v), and then re-equilibrated twice with 0.1% FA (v/v). After loading the sample onto the columns, they were washed once with 0.1% FA (v/v). The peptides were then eluted using 80% ACN/0.1% FA (v/v). The washing and re-equilibration volumes for the 3-cc column were 2 ml, while for the 1-cc columns, 1 ml was used. For the elution step, 1 ml or 600 μl was used depending on the column size. The eluates were then dried by vacuum centrifugation and stored at −80 °C until further use.

### Digestion Efficiency

HeLa cells were lysed, and proteins reduced and alkylated as described above. The samples were subsequently diluted to final concentrations of 50 mM ammonium bicarbonate (ABC), 0.24% (w/v) sodium deoxycholate (SDC), and either 0% or 6% (v/v) acetonitrile (ACN). Digestion was performed by adding trypsin or GluC (Promega) at a 1:50 enzyme-to-substrate ratio followed by incubation overnight at 37 °C with continuous shaking at 700 rpm. Digestion was stopped by acidification with neat FA to a final concentration of 5% (v/v). SDC and other insoluble matter were removed *via* centrifugation at 20.000*g* and 4 °C for 10 min. As a final step, peptides were desalted using C18 StageTips (Empore) and eluted with 60% ACN/0.1% FA (v/v). The eluates were then dried by vacuum centrifugation and stored at −80 °C until further use.

### Preparation of Phosphonate-Tagged Internal Peptides

For initial method optimization, HeLa tryptic digest was resuspended in 200 mM HEPPS, pH 8.5 and mixed with varying amounts of 1-(6-Phosphonohexanoyloxy)-2,5-pyrrolidinedione (NHS-PnTAG, MedChemExpress, HY-28043), maintaining a final ACN concentration of 20% (v/v), and incubated for 1 h at 20 °C in a thermoshaker with shaking at 500 rpm. Respective volumes and concentrations are specified in the results section.

For the multiplexing and label-free experiments, desalted peptides were reconstituted in 200 mM HEPPS (pH 8.5) to a concentration of 10 μg/μl and incubated for 10 min at 20 °C while shaking at 500 rpm. The free amino groups of the internal tryptic peptides were phosphonate-tagged by adding NHS-PnTAG at a final concentration of 13.7 mM in 20% ACN. The reaction was incubated for 1 h at 20 °C and 500 rpm. To quench the tagging reaction, 100 μl of a 75 mM ABC solution was added to the mixture and incubated for 30 min at 25 °C at 500 rpm. Finally, water was added to reduce the ACN concentration to 4% (v/v), and samples were acidified with FA to 5% (v/v). The peptides were desalted as previously described.

### High-pH Reversed-Phase Fractionation

TMT-labeled and phosphonate-tagged peptides were subjected to offline basic pH reversed-phase (HpH RP) fractionation. Peptides were reconstituted in 25 mM ammonium bicarbonate at pH 8.5 and loaded onto an XBridge BEH C18 column (Waters; 3.5 μm, 4.6 mm × 250 mm) connected to a Dionex Ultimate 3000 HPLC system (Thermo Scientific). Peptides were loaded for 3 min at 100% buffer A (95% H_2_O, 5% ACN, 2.5 mM ABC, pH 8.5), and separated at a flow rate of 1 ml/min using a 42.5-min linear gradient from 7% to 45% of buffer B (5% H_2_O, 95% ACN, 2.5 mM ABC, pH 8.5), followed by a 3.5-min washing with 85% BA total of 96 fractions were collected, which were subsequently pooled into 24 fractions, acidified with FA to a final concentration of 0.5%, and dried by vacuum centrifugation at 45 °C.

### Depletion of Phosphonate-Tagged Peptides

Phosphonate-tagged and fractionated peptides were depleted using 5 μl Fe(III)-NTA cartridges in an automated fashion on the AssayMAP Bravo Platform (Agilent Technologies), adapting a standard enrichment protocol (31). In detail, cartridges were primed at a flow rate of 300 μl/min with 150 μl of priming buffer (0.1% TFA in ACN) and equilibrated at 10 μl/min with 150 μl of loading buffer (0.1% TFA in 80% ACN). Subsequently, peptides, dissolved in 200 μl of loading buffer, were loaded at 5 μl/min onto the cartridge, and the flow through was collected into a separate plate. Columns were washed with 150 μl of loading buffer at a flow rate of 10 μl/min, and the flow through was collected and combined with the previous one. Finally, bound peptides were discarded with 60 μl of 1% NH_4_OH (v/v). The depleted fractions were subsequently pooled into 12 fractions.

For label-free experiment, depletion was performed *via* solid-phase extraction using 30 mg, 1cc zirconia-based HybridSPE cartridges (Sigma), which provide a higher binding capacity required for a 200 μg input. The columns were first conditioned with 500 μl of loading buffer (1% FA in 80% ACN). Afterward, samples were loaded in 250 μl of loading buffer, and the flow throughs were collected. Cartridges were sequentially washed twice with 400 μl of 1% FA in 50% ACN and 400 μl of 1% FA in 10% ACN. During all washing steps the flow through were collected, combined with the previous ones, and then dried by vacuum centrifugation at 45 °C. Finally, depleted peptides were desalted using SDB-RPS StageTips (Empore) *via* centrifugation at 100 to 500*g* for 2 to 5 min. To this end, peptides were reconstituted in 1% TFA and transferred to StageTips. After loading the samples, the StageTips were washed sequentially with 100 μl of 1% TFA in IPA, 10 mM NH_4_FA in 50%ACN/5% FA, and 0.2% TFA. Peptides were eluted using 150 μl of 1% NH_4_OH in 80% ACN. The SDB-RPS StageTips cleanup procedure was also used to desalt samples of the titration experiments. All depleted samples were dried down at 45 °C and stored in −20 °C until LC-MS/MS analysis.

### Nanoflow LC-MS/MS

The analysis of N-terminal peptides (depletion flow-through) was performed either on a Dionex Ultimate 3000 UHPLC+ coupled to an Orbitrap Eclipse mass spectrometer or on a Vanquish Neo UHPLC system coupled to an Orbitrap Exploris 480 mass spectrometer (all Thermo Fisher).

The Dionex system was equipped with a 2 cm trap column (75 μm i.d., packed in-house with 5 μm Reprosil C18 beads; Dr Maisch) and a 45 cm analytical column (75 μm i.d., packed in-house with 1.9 μm ReproSil-Pur C18-AQ beads; Dr Maisch). Trapping and washing were performed at 5 μl/min for 10 min with solvent A (0.1% FA in water). Subsequently, peptides were transferred to the analytical column at 300 nl/min in a total analysis time of 90 min with a gradient of 8%-34% (v/v) solvent B (0.1% FA, 5% DMSO in ACN) in 80 min. The mass spectrometer was operated in a data-dependent MS3 mode. Every 3 s, a MS1 survey scan was recorded from 360 to 1500 m/z with a resolution of 60k in the Orbitrap. The MS1 AGC target was set at 100%, and the maxIT was set to 50 ms. Precursors were targeted for HCD fragmentation with an NCE of 34% if the charge was between 2 and 6, and the intensity exceeded 2e4. The quadrupole isolation window was set to 0.7 Th. The MS2 spectra were acquired in the Orbitrap with a resolution of 15K, an AGC target at 100%, and maxIT of 22 ms. Precursors that have been targeted for fragmentation were excluded for 90 s for all possible charge stages. TMT reporter ions were measured in a consecutive MS3 scan based on the previous MS2 scan. Thus, a new batch of precursor ions was isolated with a quadrupole isolation window of 1.2 Th. The isolated precursor was then HCD-fragmented identically to the previous MS2 scan. The top 10 fragment ions of the MS2 scans were isolated in the ion trap in parallel *via* synchronous precursor selection, and HCD fragmented with an NCE of 55%. The MS3 spectrum was acquired with 30k resolution from 100 to 500 m/z in the Orbitrap, and the phase-constrained spectrum deconvolution method (PhiSDM) was enabled for the TMT reporter ions. The MS3 AGC target was set at 500%, and the maxIT was set to 54 ms.

The Vanquish Neo system was equipped with a 5 cm trap column and a 40 cm analytical column, both packed in-house as described above. Sample loading was performed using 0.1% FA at a flow rate of 5 μl/min, then peptides were transferred to the analytical column and separated at 300 nl/min using an 81-min linear gradient 4% to 25% of solvent B (0.1% FA, 3% DMSO in ACN) and solvent A (0.1% FA, 3% DMSO in water). The total analysis time was 90 min.

The Orbitrap Exploris 480 was operated in data dependent and positive ionization mode. MS1 spectra were recorded in the Orbitrap from 360 to 1350 m/z at a resolution of 60K, with an AGC target value at 100%, and maxIT of 50 ms. The top 25 precursor ions in the MS1 scans with a charge between 2 and 6 were isolated *via* the quadrupole with an isolation window of 0.7 Th, and HCD-fragmented with a NCE of 36%. MS2 spectra were recorded in the Orbitrap at 15K resolution, with an MS2 AGC target value at 200%, and maxIT set to 22 ms. The PhiSDM option was enabled, and dynamic exclusion was set to 40 s.

Labeling and tagging efficiency samples were acquired on the Vanquish-Exploris system, with the following modifications: MS1 spectra were recorded from 360 to 1300 m/z with an AGC target value at 300%, the top 15 precursor ions in the MS1 were subjected to HCD fragmentation with an NCE of 30%, quadrupole isolation window was set to 1.3 Th, and MS2 spectra were recorded at 15K resolution with an AGC of 200% and maxIT set to 22 ms, Dynamic exclusion was 30 s.

Label-free samples were acquired on the Vanquish-Exploris system, with the following modifications: MS1 spectra were recorded from 400 to 1500 m/z with an AGC target value at 300%, the top 15 precursor ions in the MS1 were subjected to HCD fragmentation with a stepped NCE of 35 ± 5%, quadrupole isolation window was set to 1.3 Th, and MS2 spectra were recorded at either 15K of 30K resolution with a maxIT set to 22 or 54 ms, respectively. Dynamic exclusion was 30 s.

Samples for digestion-efficiency evaluation were acquired on the Vanquish-Exploris system, with the following modifications: a 51-min linear gradient 4% to 25% of solvent B, a total analysis time of 60 min, an MS1 resolution of 60K, an MS1 AGC target value of 100% and a dynamic exclusion of 25 s.

### Data Analysis

Raw mass spectrometry data were processed with the FragPipe (32) software (version 22.0). Spectra were searched against the UniProtKB database (Human, UP000005640, 104,726 entries including isoforms, downloaded on 10.2023). Enzyme specificity was set to semi-trypsin_r with free N-terminus, allowing for two missed cleavages, and the search included cysteine carbamidomethylation as a fixed modification and methionine oxidation as a variable modification. TMT (+229.16293) or DMA-PEG3ylation (+231.14706) were fixed modifications on peptide N-terminus and lysine. To identify acetylated protein N-termini, separate database searches were performed in which TMT (+229.16293) or DMA-PEG3ylation (+231.14706) were set as variable modifications on peptide N-terminus together with protein N-terminal acetylation (+42.0106).

Additional separate searches were conducted to evaluate labeling efficiency. Thus, TMT (+229.16293) or DMA-PEG3ylation (+231.14706) was specified as a variable modification on primary amines (lysine and protein N-terminus) or as a fixed modification on primary amines and, additionally, as a variable modification on serine, threonine, and tyrosine. Enzyme specificity was set to strict_trypsin, and up to three or two missed cleavages were allowed, for labeling and overlabeling search, respectively. For tagging efficiency, 6-Phosphonohexanoylation (+178.03949) was specified as a variable modification on lysine and peptide N-terminus or as a fixed modification on primary amines and as a variable modification on serine, threonine, and tyrosine. For depletion efficiency evaluation, TMT or DMA-PEG3ylation was specified as a fixed modification on primary amines, while 6-Phosphonohexanoylation was specified as a variable modification on peptide N-terminus. To account for peptides carrying 6-Phosphonohexanoylation instead of the fixed label, mass shifts of −51.12344 Da (relative to TMT) or −53.10757 Da (relative to DMA-PEG3ylation) were used.

For digestion-efficiency assessment, database searches were performed with enzyme specificity set to either strict_trypsin or GluC under bicarbonate conditions (gluc_bicarb), allowing up to three missed cleavages.

The precursor and fragment mass error tolerances were each set to 20 ppm, while the precursor mass tolerance for the open-search analysis was configured to range from −150 Da to +500 Da.

For TMT multiplexing and digestion-efficiency data, peptide-to-spectrum matches (PSM) rescoring *via* MSBoster (33) was enabled, using predictions from Prosit *via* the Koina service (34). Finally, identifications were adjusted to 1% false discovery rate (FDR) with percolator (35) and Philosopher (36), integrated into FragPipe.

Data analysis was performed with the Perseus (37) software (version 2.1.3.0). Peptide identifications were filtered to remove contaminants before performing data normalization of the log2-transformed peptide intensity values by median centering, as implemented in Perseus. For statistical analysis, only peptides quantified in at least three biological replicates in at least one experimental group were retained. Missing values were imputed from a normal distribution in Perseus using default parameters (width = 0.3, down shift = 1.8, mode = separately for each column), but only for peptides that did not already meet this threshold (3 valid values) in both groups. Differential analysis (STS *versus* Mock) was conducted using Welch’s *t* test with a permutation-based FDR threshold of 5% to account for multiple testing. Significant hits were further required to exhibit an absolute fold-change of at least 2.

### Experimental Design and Statistical Rationale

For protocol development, we sought to find optimal conditions for primary amine blocking or tagging and focused on peptide population analysis in individual samples. All experiments were conducted as three independent experiments using single replicates per condition. For method optimization, individual channels of TMT10plex reagent were used independently, and varying amounts of reagents were mixed with varying amounts of cell lysate. All samples were analyzed using label-free single-shot LC–MS/MS data-dependent acquisition runs. Reactions efficiency as well as over-derivatization in each sample was calculated based on summed modified peptide intensities, extracted from the *combined_ion.tsv* table. Depletion efficiency was calculated based on summed modified peptide intensities or counts, extracted from the *combined_modified_peptide.tsv* table.

For benchmarking, TMT and LFQ experiments were performed with five biological replicates in each experimental condition (Mock or STS). The differential significance between sample groups was calculated using two sample Welch’s *t* test and the threshold was set as q-value <0.05 and fold-change >2.

## Results and Discussion

### Workflow Optimization

We initially sought to evaluate the feasibility of performing primary amine blocking directly on the proteome lysate, immediately followed by digestion, without requiring any cleanup step. SDC has emerged as a widely used detergent in proteomics by virtue of enhancing trypsin activity and being easy to remove prior to LC-MS analysis (38). Given that this detergent lacks a primary amine group and is soluble in both polar and non-polar solvents, we reasoned that it could be used effectively for cell lysis and in a one-pot blocking/digestion procedure, where the TMT reaction and digestion share a common buffer. To test this hypothesis, a protein and TMT titration experiment was performed in triplicates using a fixed protein amount of 100 μg in varying volumes of lysis buffer (1 or 2% SDC, 200 mM HEPPS, pH 8.5) ranging from 50 to 75 μl, and different amounts of TMT (200 and 400 μg) in 20% ACN. The reaction was stopped by adding ABC to a final concentration of 50 mM, also lowering the SDC (0.24%) and ACN (6%) concentrations for direct trypsin digestion. Samples were analyzed using single-shot LC-MS/MS runs (Supplemental Table S1). Across the entire range of tested protein and TMT quantities, at least 98% of the detected peptides bearing labelable amines (protein N-termini and lysine side chains) were fully labeled (Fig. 1*A*). As expected, the higher TMT-to-protein ratio resulted in higher labeling efficiency; however, the magnitude of this difference is negligible. Consequently, few unlabeled or partially labeled peptides were observed. Interestingly, the corresponding over-labeling analysis revealed that on average, just ∼6% of the detected peptides contained at least one TMT-labeled serine, threonine, or tyrosine residue (Supplemental Fig. S1*A*), with minimal difference across the various TMT concentrations.

**Fig. 1:**
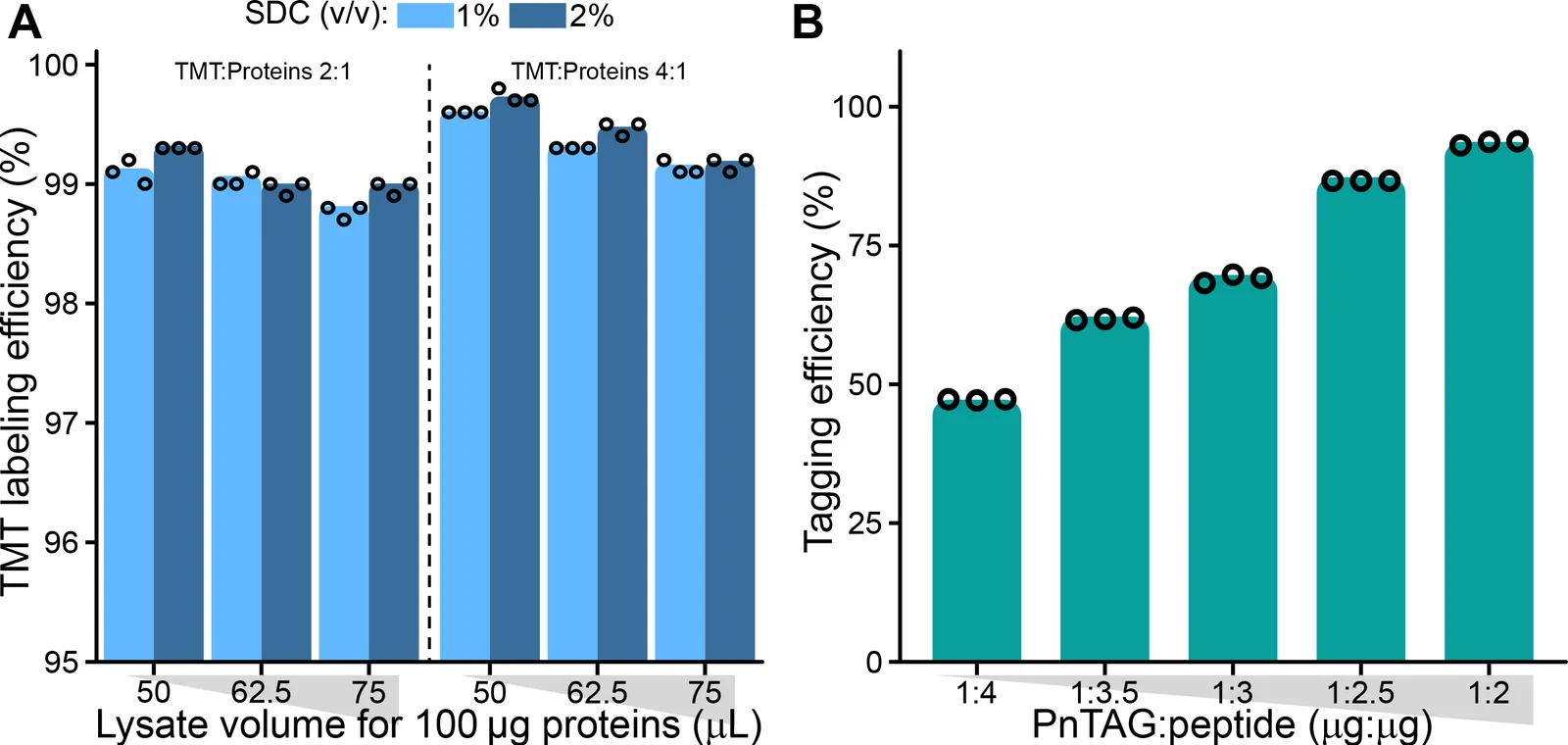
**Evaluation of protein-level TMT labeling and peptides phosphonate-tagging protocols.***A*, Bar plot of the percentage of peptide intensity from labeled peptides at lysines and protein N-termini (labeling efficiency) across different reaction volumes and amounts of TMT10 reagent. *B*, Bar plot showing the proportion of HeLa peptides (by intensity) correctly tagged at lysines and peptide N-termini across different amounts of NHS-PnTAG reagent. For all plots, bars are drawn at the mean value (n = 3), and dots indicate individual replicates.

While trypsin remains the gold-standard protease in bottom-up proteomics and N-terminomics due to its high specificity and digestion efficiency, it is well established that tryptic digestion does not provide complete coverage of all protein regions (39). Consequently, the use of complementary proteases can reveal N-termini that are inaccessible to trypsin alone. Although it has been reported that most proteolytic enzymes remain active at low concentrations of SDC (40, 41, 42), we nevertheless evaluated whether GluC, selected because of its high specificity and widespread use in N-terminomics (43) remains active under the conditions established for direct primary amine blocking and subsequent digestion. Like trypsin, GluC showed no reduction in digestion efficiency under our experimental conditions (Supplemental Fig. S1*B* and Supplemental Table S1). Given that the residual SDC and ACN concentrations can be further reduced by dilution if required, the workflow provides flexibility for the incorporation of alternative proteases.

Taken together, this indicates that blocking of primary amine directly on the cell lysate is possible, greatly facilitating the sample preparation and sidestepping variable sample losses due to multiple cleanups. We note that the abundance of primary amines varies significantly depending on the sample type, including contributions from metabolites, lipids, free amino acids, and so on; hence, to ensure complete labeling (>99%), while minimizing overlabeling, we identify 3:1 as a suitable TMT-to-protein ratio for subsequent experiments.

### NHS Ester-Mediated Phosphonate Tagging of Internal Peptides

Removal of internal peptides is essential in N-terminomics to specifically identify peptides originating from the N-terminus of proteins or newly exposed N-termini after proteolysis. This is often achieved through a Schiff base reaction, where the α-amines of internal peptides react with a reagent that alters their hydrophobicity or molecular weight (44), allowing them to be captured and removed. While the reaction is selective, it generally requires several hours to ensure complete derivatization. Additionally, it requires careful handling due to the potential hazards of the reagents or byproducts involved. McDonald *et al*. previously reported the successful use of NHS chemistry for the efficient removal of internal peptides (19, 45). Here, we adapted the PTAG approach (29) by replacing phosphate tagging through reductive amination with direct phosphonate tagging. Inspired by a strategy based on crosslinking peptides with a phosphonate moiety using NHS chemistry (46), we phosphonate-tagged internal peptides using an inexpensive and fast-reacting NHS ester. Depletion is then seamlessly performed using metal-based coordination methods that are broadly used for phospho-peptide enrichment (47, 48). We note that, while the non-MS-cleavable crosslinker PhoX could in principle be used for this purpose, its application is less practical owing to the challenges associated with reaction stoichiometry and titration. In addition, the reagent is relatively expensive when obtained at the high purity required for such application.

To evaluate the efficiency of our tagging strategy, we used a HeLa tryptic digest and performed again a titration experiment using a fixed amount of peptides and increasing concentration of NHS-PnTAG, resulting in tag-to-peptide ratios of 1:4 up to 1:2 (Fig. 1*B* and Supplemental Table S2). Single-shot LC-MS/MS analysis revealed nearly complete derivatization at a 1:2 ratio, with minimal over-tagging on serine, threonine, or tyrosine residue (Supplemental Fig. S1*C*). To rule out the formation of other unexpected products due to minor impurities in the NHS-PnTAG reagent (purity >95% according to the manufacturer), we used the open search functionality of MSFragger to detect any new modifications on the peptides (Supplemental Table S2). Although we identified over 40 potential mass shifts, they only accounted for ∼15% of the identified peptides, and the majority of these were also present in the control experiments (Supplemental Fig. S1*D*).

### TMT-SIMPATICO Performance in Identifying Proteolytic Events in Apoptosis

After establishing a streamlined protocol for primary amine blocking and internal peptides tagging, we assessed the ability of SIMPATICO to identify proteolysis-generated termini (Fig. 2*A*). To achieve this, we induced caspase-mediated apoptosis in Jurkat T-cells by treating them with the pan-kinase inhibitor Staurosporine (STS) for 6 hours. The experiment included five replicates of STS-treated samples and five replicates of DMSO-treated controls (Mock). Cells were lysed in SDC buffer, and primary amines were labeled with TMT10. The combined proteomes underwent direct overnight trypsin digestion and subsequent desalting. Internal peptides were tagged with phosphonate as described above, followed by another step of desalting, HpH RP fractionation, and automated IMAC-based depletion. The number of fractions was chosen based on the theoretical molarity (30) of phosphonate-tagged molecules (∼38 nmol/100 μg of input material) and the binding capacity of the IMAC cartridges (∼58 nmol), as specified by the manufacturer, to maximize the capture of tagged peptides while minimizing cartridge overloading. To evaluate how effectively this strategy selected N-terminal peptides and depleted internal peptides, we next assessed the depletion efficiency of the workflow. The analysis revealed a sample purity of ∼53% based on peptide intensities and ∼74% based on peptide counts across the fractions, indicating that highly abundant internal peptides were not completely depleted. However, based on NanoDrop measurements of the purified material and the recorded MS signal intensities, we estimated a recovery of roughly 10 to 15 μg per fraction. Assuming a conservative average sample purity of ∼60% and a starting amount of 10 × 200 μg of material, the depletion efficiency was estimated to exceed 99%. As a result, we identified over 22,000 N-terminal peptides from approximately 4500 proteins across all biological replicates (Supplemental Table S3), with the majority of peptides being consistently quantified across all samples (Supplemental Fig. S2*A*). Importantly, the quantitative reproducibility between the replicates was high (Supplemental Fig. S2*B*), with an average Person correlation within conditions of above >0.95.

**Fig. 2:**
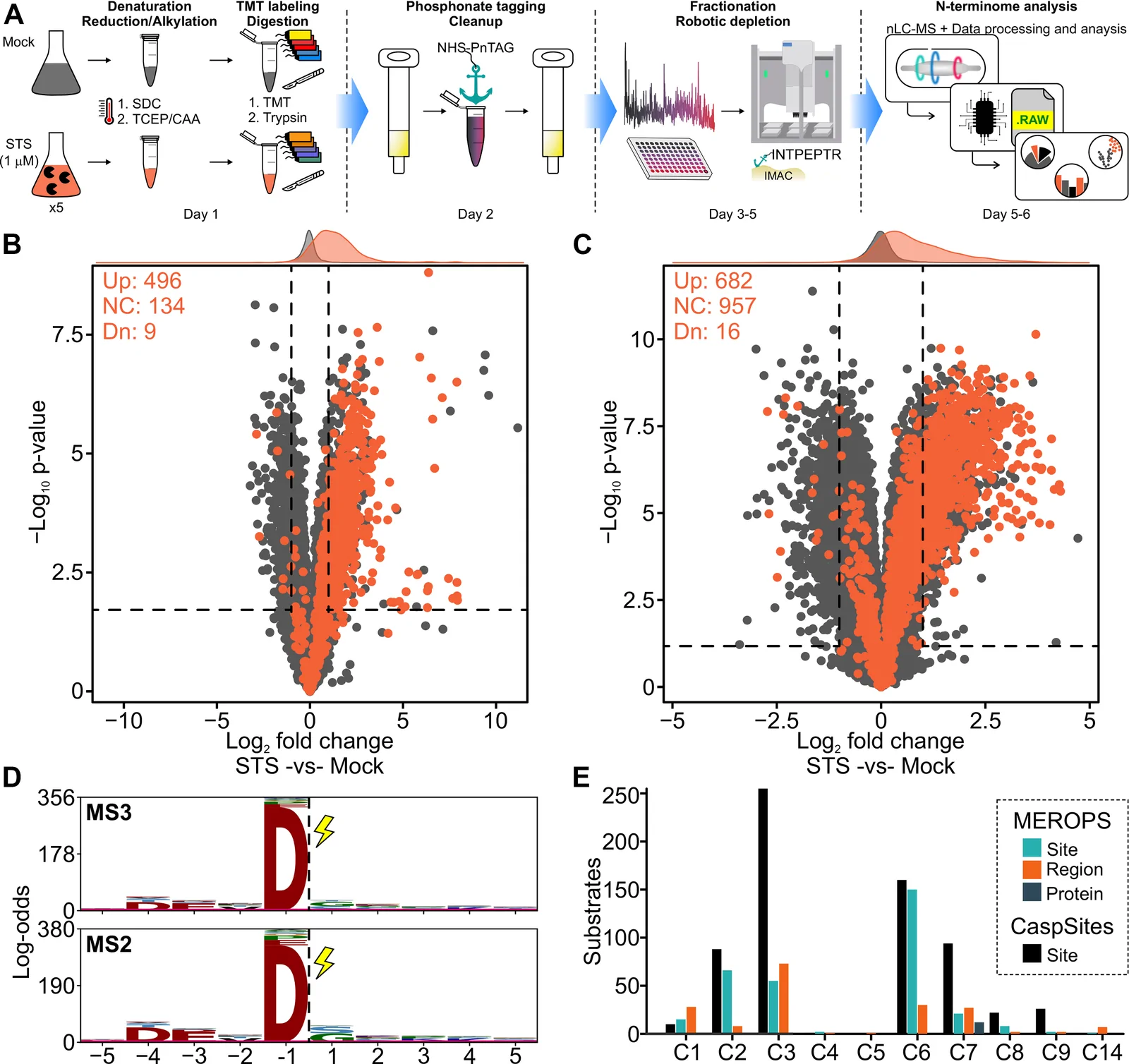
**Protocol for streamlined identification of N-terminal peptides.***A*, experimental stages and timing for the TMT-SIMPATICO workflow for the identification of caspase substrates: Protein samples are denatured, reduced, and alkylated. 200 μg of proteins per sample are directly labeled with 600 μg TMT and afterward combined and digested with trypsin (day 1). The resulting peptides are desalted *via* a C18 cartridge before and after internal peptide tagging with NHS-PnTAG (day 2). The peptide mixture is fractionated *via* basic pH reverse-phase chromatography, and N-terminal peptides are recovered by negative selection using an automated Fe(III)-IMAC workflow to deplete PnTAG-peptides (days 3–5). N-terminal peptides and cleavage events are identified and quantified *via* nLC-MS/MS (days 5–6). *B*, Volcano plot (Welch’s *t* test) showing the N-termini enriched in STS-treated *versus* Mock-treated Jurkat cells. N-termini generated by cleavage of C-terminal to aspartic residues are highlighted in orange. The *x-axis* shows log2 fold change, while the *y-axis* shows -log10 *p*-value adjusted for multiple hypothesis testing using the Permutation-based FDR procedure. Dashed lines are at 5% FDR and log2 fold change of ± 1. Results are from five biological replicates for each condition. Each dot corresponds to an N-terminal peptide. Peptides derived from aspartic acid cleavage are shown in *orange*. For aspartic acid cleavages, the number of upregulated (Up), non-changing (NC), and downregulated (Dn) events is indicated. *C*, same as in (*B*) but from the MS2-based acquisition. *D*, pLogo plots of the enriched cleavages (>2-fold STS-vs-Mock, FDR <5%) in response to the STS treatment. Letters larger than the significance threshold (*red line*) are deemed significantly enriched in those positions. *E*, Bar chart showing the potential upstream caspase protease responsible for the generation of the significantly enriched cleavages C-terminal of Asp upon STS treatment, according to the MEROPS and Caspsites databases. For MEROPS we defined three different categories describing the information obtained about the cleavage position: explicit cleavage sites (*teal*), within (*orange*), or outside (*grey*) the protein region reported in the database.

Since the number of N-terminal peptides largely depends on the proteome's degradation state under investigation, we first benchmarked our approach against recently established methods for native protein N-termini identification (24, 49, 50). Our analysis resulted in the identification of 380 canonical unmodified protein N-termini, with or without the initiator methionine excision, and additionally 117 isoforms protein N-termini. When protein N-terminal acetylation was included as a variable modification in the search 1076 unique acetylated protein N-termini were identified. Of those, 902 contained a TMT-labeled lysine and therefore quantified, resulting in a total of 1019 non-redundant native N-termini. We further explored the list of identified N-termini and compared it with protein processing annotations available in UniProt. This analysis identified 355 annotated maturation-derived N-termini arising from translation initiation or protein processing events, such as signal, transit, and propeptide removal. Because the precise position of mature protein N-termini can vary by a few residues due to uncertainties in annotation, biological heterogeneity of processing events, or limitations in the experimental determination of cleavage sites, we also applied a relaxed matching criterion allowing N-termini to fall within ±3 (or ±5) amino acids of the annotated position. Under these criteria, the number of matched maturation-derived N-termini increased by 227 and 208, respectively. We further extended our analysis to alternative translation initiation sites using sequences deposited in OpenProt, a proteogenomics resource that catalogs alternative open reading frames and protein isoforms (51). This analysis identified an additional 603 protein N-termini derived from alternative protein isoforms.

Notably, the predominance of unannotated N-termini is in agreement with previous N-terminomics studies (50), which have similarly reported that a substantial fraction of experimentally detected N-termini cannot be explained by current protein annotation databases. This observation likely reflects both incomplete annotation of protein maturation events and the existence of previously uncharacterized translation initiation and proteolytic processing events, as well as apoptosis-driven protein degradation following STS treatment.

Although a direct comparison is not possible due to differences in sample types as well as variations in MS instrumentation, input material, sample preparation, and data processing, the values observed here are consistent with previously reported results, demonstrating the capability of SIMPATICO for robust N-terminomics analysis. Notably, the median coefficient of variation (CV) of the quantified N-blocked peptides ranged from 18.1% (Mock) to 17.6% (STS), matching or surpassing those reported for recently established methods (Supplemental Fig. S2*B*). The low variability likely results from the reduced sample handling in our workflow, which eliminates cleanup steps prior to multiplexing.

Principal component analysis (PCA) revealed a clear separation between Mock and STS-treated samples (Supplemental Fig. S2*C*), indicating distinct proteolytic profiles.

We next performed two-sample *t*-tests to identify apoptosis-induced cleavage events by comparing STS-treated and Mock-treated cells. Significantly upregulated peptides were stringently selected based on a >2-fold increase and a *q*-value <0.05. In total, SIMPATICO identified 908 cleavage sites that were significantly enriched upon STS treatment (Fig. 2*B*). Notably, aspartyl cleavages, characteristic of caspase-mediated proteolysis, were also consistently quantified across all samples (Supplemental Fig. S2*D*), but prominently enriched in STS-treated samples with 496 unique sites, of which 490 were non-redundant, consistent with activation of apoptotic pathways. Interestingly, several N-terminal peptides show a negative fold change, appearing less abundant in the STS-treated condition, suggesting these N-termini were lost due to cleavage further downstream toward the C-terminus, although the exact cleavage site cannot be pinpointed.

Analyses of the amino acid composition surrounding the 490 cleavage sites clearly showed a preference for small residues such as serine and glycine c-terminal to the cleaved aspartate, as well as the overrepresentation of the DEVD| consensus motif, a well-documented preference of several caspases (52, 53).

Motivated by the high number of cleavage events detected, we sought to further deepen the coverage of the N-terminome by employing a more rapid acquisition method.

To this end, we reanalyzed the same samples using a faster MS2-based acquisition for TMT quantification (54). This approach enabled the identification of an additional 30,242 N-terminal peptides with an overlap of ∼15.553 to the MS3 acquisition, thereby expanding. the cumulative dataset by 1842 native protein N-termini, and 390 STS-induced aspartyl cleavages (Fig. 2, *C* and *D*, and Supplemental Table S4). Similarly, the MS2-based measurements exhibited high reproducibility and robustness, as evidenced by the tight clustering of biological replicates and the clear separation between Mock- and STS-treated samples in PCA space (Supplemental Figs. S2*C*, S3, *A* and *B*).

To generate a list of known caspase substrates for comparison, we compared the STS-induced aspartyl cleavages detected with SIMPATICO to the MEROPS and CaspSites databases (55, 56). This analysis showed that 507 (57%) of the identified substrates were previously recognized as either verified caspase substrates with exact cleavage sites match or as potential substrates with cleavage evidence within the relevant sequence region or at the protein level (Fig. 2*E*). As expected, given their reported activation by STS (57, 58), CASP-3 and -6 emerged as the most active caspases. The remaining 402 of candidates, on 339 proteins, represented yet unreported novel substrates.

Besides potential caspase substrates, our analysis identified STS-induced cleavages at several residues other than aspartate, possibly the result of proteasomal degradation or additional proteolytic events. Notably, 92 glutamyl cleavages (∼5%) were enriched in the STS-treated sample. Closer inspection of the sequence surrounding the cleavage site revealed the preference for the DEVE motif (Supplemental Fig. S3*C*), which has been indicated to be cleaved by apoptotic CASP-3 and -7, suggesting that also these proteolytic events are likely mediated by these executioner caspases during apoptosis. In addition, we identified 16 known substrates of cathepsins, lysosomal proteases for which there is growing evidence of activation, although to an unknown extent, during apoptosis (59), highlighting the strength of SIMPATICO in capturing proteolytic events with high sensitivity across the N-terminome.

### LFQ-SIMPATICO

Quantitative N-terminomics is currently mostly limited to multiplexed quantification methods such as SILAC, dimethyl labeling, or TMT/iTRAQ (20). Techniques like COFRADIC and its derivatives, although effective for N-terminal enrichment, often require extensive fractionation, which typically restricts their use to the analysis of only a few samples at a time (60). Thus, while multiplexing enables relative quantification, it inherently limits the number of samples that can be analyzed simultaneously. Scaling beyond the multiplexing capacity requires revising the experimental design to include a common reference sample across multiplexing sets (61), which could become impractical. To date, high-throughput, label-free N-terminomics remains largely unexplored. To address this gap, we investigated the feasibility of adapting our SIMPATICO workflow into a label-free approach. One of the primary challenges in this adaptation is the efficient blocking of primary amines directly in the lysate. Although Native TMT (TMTzero) can be used for this purpose, its high cost makes it unsuitable for large-scale analyses. NHS-acetate, commonly used in COFRADIC-like protocols, also presents limitations: acetylation of primary amines can suppress peptide charge states (62), as the acetylated nitrogen can no longer retain a proton, thereby hindering MS-based identification of N-terminal peptides. To overcome these limitations, we explored commercially available NHS-based derivatization reagents that would not lead to charge suppression. Candidates such as DMABA NHS-ester and RapiFluor-MS include a tertiary, protonable amine group (63, 64), but both have poor water solubility. We turned our attention to PEG-based NHS esters, a class of amine-reactive reagents with improved water solubility commonly used in click chemistry. We identified DMA-PEG3-NHS as a promising candidate for primary amine blocking (Fig. 3*A*). This reagent contains a tertiary amine as a protonable site and a polyethylene glycol linker for improved solubility in water. Titration experiments demonstrated that DMA-PEG3-NHS can effectively replace TMT for primary amine blocking without compromising peptide ionization or identification.(Fig. 3*B*, Supplemental Fig. S4, *A* and *B*, and Supplemental Table S5). Building on this, we evaluated the feasibility of a label-free version of the SIMPATICO workflow to identify proteolysis-generated N-termini.

**Fig. 3:**
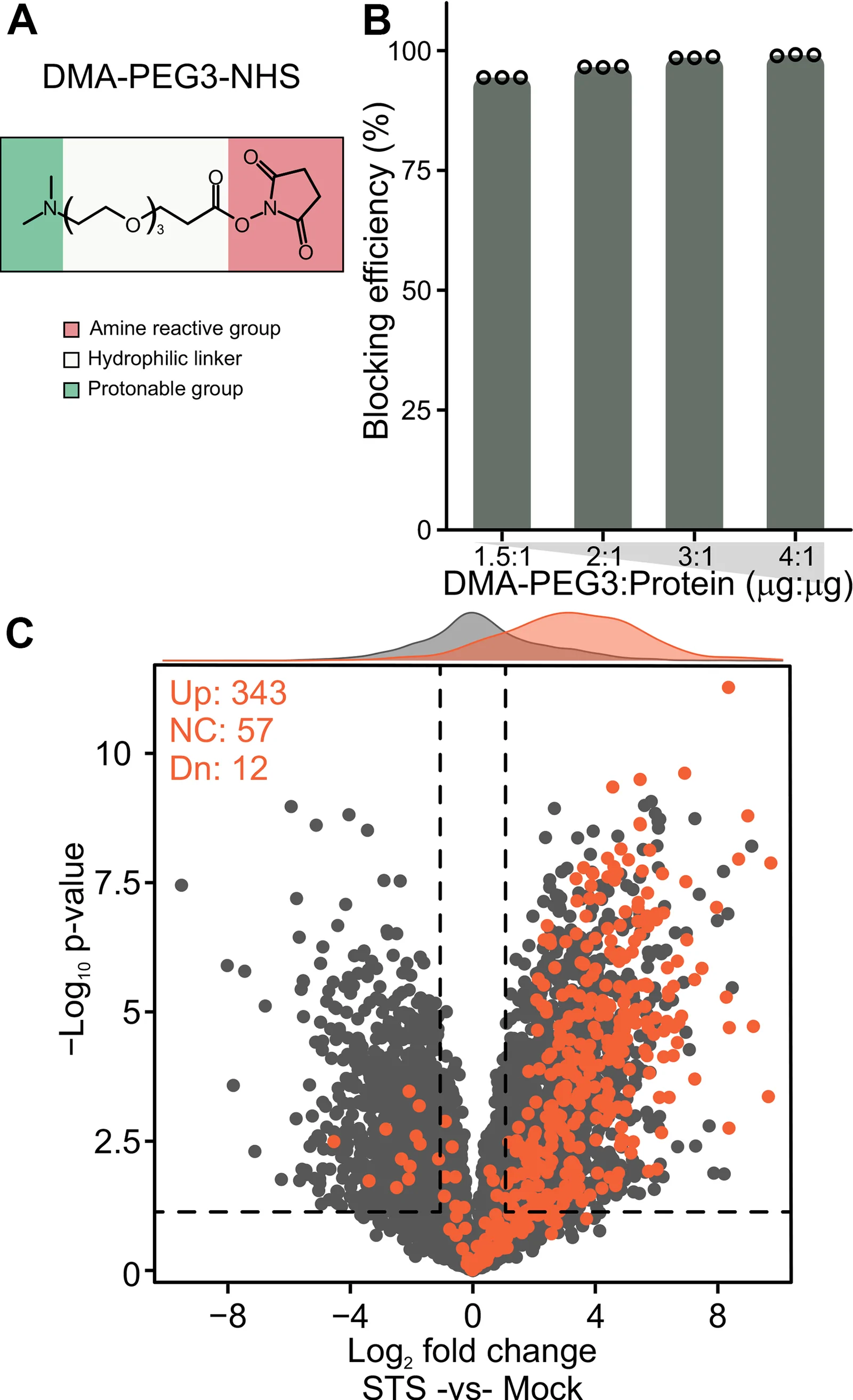
**N-terminomics *via* label-free quantification.***A*, chemical structures of DMA-PEG3-NHS. *B*, Bar plot showing the proportion of HeLa peptides (by intensity) correctly blocked at lysines and protein N-termini (blocking efficiency) across different amounts of DMA-PEG3-NHS reagent. Bars are drawn at the mean value (n = 3), and dots indicate individual replicates. *C*, same as in Figure 3*B*, but from the label-free experiment. Dashed lines are at 5% FDR and log2 fold change of ± 1. Results are from five biological replicates for each condition. Each dot corresponds to an N-terminal peptide. Peptides derived from aspartic acid cleavage are shown in *orange*.

As in previous experiments, Jurkat cells treated with either STS or DMSO were lysed in SDC buffer, and primary amines were blocked using DMA-PEG3-NHS directly in the lysate. Upon tryptic digestion and desalting, internal peptides were selectively tagged with NHS-PnTAG and removed using a zirconia-based SPE. The flow-through from this step was further purified with SDB-RPS, leveraging the dual-mode properties of the sorbent to wash away any undepleted internal peptides from the previous step, which predominantly appear as +1 charge state species due to the acylation of the peptide N-terminus.

The enriched N-terminal peptide samples were analyzed *via* label-free nLC-MS/MS. The high binding capacity of the zirconia-based SPE cartridge yielded an average sample purity of >99.9%, corresponding to near-complete depletion of internal peptides. Using this approach, we identified 5598 N-terminal peptides, including 412 generated by cleavage C-terminal to aspartic residues (Supplemental Table S6).

The lower number of detected N-terminal peptides was expected. In the label-free experiments, no peptide fractionation was performed, meaning that sample complexity remained high and limited the depth of N-terminome coverage. Consequently, in the absence of multiplexing, samples were analyzed individually rather than pooled. This prevented cumulative signal enhancement, so the label-free approach likely captured only the most abundant N-terminal peptides (Supplemental Fig. S4*C*). Furthermore, peptide identifications were not rescored using MSBooster, as the dimethylaminoPEG3ylation is not currently supported for rescoring by predictive models, which leaves room for further improvement in peptide identification. Future efforts could include the use of mild peptide fractionation prior to MS analysis or the adoption of data-independent acquisition (DIA), especially given that recent advances in transfer learning have enabled predictive models to generalize DIA searches to previously unseen modifications (65).

Despite these limitations, an advantage of the LFQ workflow is its ability to quantify endogenous acetylated N-terminal peptides lacking lysine residues. Because acetylated N-termini cannot be TMT-labeled and peptides without internal lysines do not carry a TMT reporter, this subset of peptides is not readily quantifiable in TMT-based workflows. When protein N-terminal acetylation was included as a variable modification in the search, 620 unique acetylated protein N-termini were reproducibly quantified at least 3 times in at least one condition, including 205 lacking lysine residues. LFQ therefore enables the virtually unrestricted quantification of acetylated N-terminal peptides, providing access to a distinct subset of the N-terminome.

Similar to the TMT-based measurements, the LFQ analysis demonstrated high reproducibility and robustness (Supplemental Fig. S4*D*), with an average Pearson correlation coefficient exceeding 0.94 across samples within each condition. The median peptide quantification CV ranged from 24.1% in Mock samples to 21.4% in STS-treated samples (Supplemental Fig. S4*E*). Consistent with the TMT data, most peptides were quantified across all samples (Supplemental Fig. S4, *F* and *G*), and principal component analysis (PCA) revealed a clear separation between Mock and STS-treated conditions (Supplemental Fig. S4*H*).

Importantly, 269 aspartyl cleavages (∼65%) were also detected in the multiplexed version of the workflow, and a clear enrichment of aspartyl cleavage events upon STS treatment was also observed (Fig. 3*C* and Supplemental Fig. S4*I*), supporting the effectiveness of the label-free approach for capturing proteolytic signatures.

## Conclusion

We have developed a streamlined sample preparation workflow that minimizes processing steps while enabling efficient and comprehensive N-terminome analysis. By combining a highly reactive NHS-ester reagent with metal coordination-based depletion, this approach allows for robust and selective capture of both canonical and neo-protein N-termini. In its label-free implementation, the workflow can easily be scaled to accommodate up to 96 samples in parallel, making it well-suited for high-throughput studies. We anticipate that this strategy will advance the study of proteolytic processes and offer deeper biological insights, potentially beyond caspase substrate identification.

## Data Availability

The mass spectrometry proteomics data have been deposited in the ProteomeXchange Consortium *via* the PRIDE partner repository (66) with the dataset identifier PXD064097.

## Supplemental Data

This article contains supplemental data.

## Declaration of Generative AI and AI-Assisted Technologies

During the preparation of this work, the authors used Grammarly and Microsoft Copilot to improve the language and readability of the manuscript. Microsoft Copilot was also used as a coding assistant during data processing and analysis. After using this tool or service, the authors reviewed and edited the content as needed and take full responsibility for the content of the publication.

## Conflict of interest

The authors declare that they have no conflicts of interest with the contents of this article.
