# Supplementary material for: SIMPATICO—Streamlined Isolation of the N-Terminome via Phosphonate Tagging and Coordination-Based Depletion
Source: Mol Cell Proteomics. 2026 Sep 10;25(10):101660. doi: 10.1016/j.mcpro.2026.101660 (PMC13673259; doi:10.1016/j.mcpro.2026.101660)
Supplement: Supplementary file 1 [file mmc1.pdf]

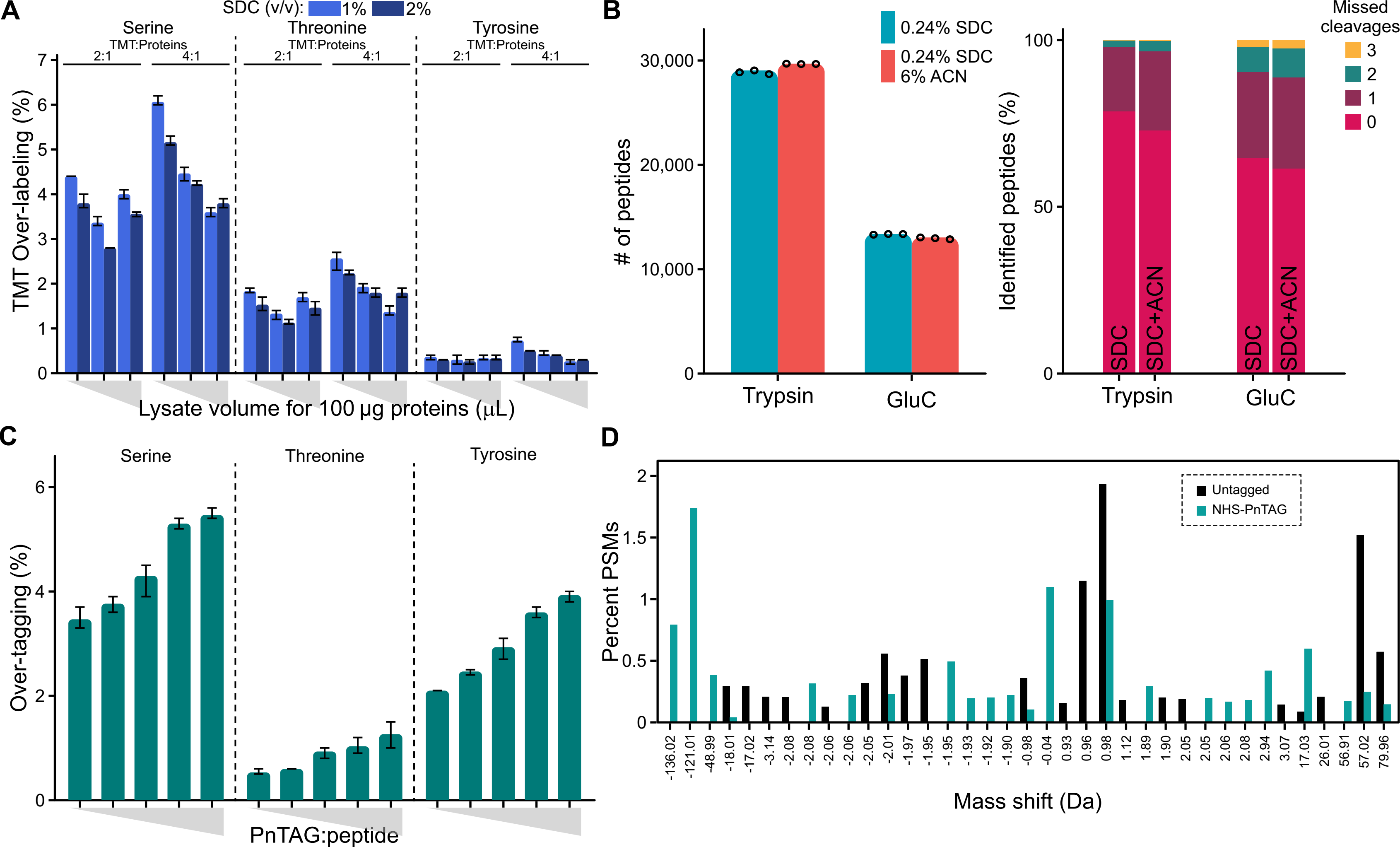
A
SDC (v/v): 1% 2%
Serine
TMT:Proteins
2:1
4:1
Threonine
TMT:Proteins
2:1
4:1
Tyrosine
TMT:Proteins
2:1
4:1
TMT Over-labeling (%)
0
1
2
3
4
5
6
7
Lysate volume for 100 µg proteins (µL)
B
0.24% SDC
0.24% SDC 6% ACN
# of peptides
0
10,000
20,000
30,000
Trypsin
GluC
Missed cleavages
3
2
1
0
Identified peptides (%)
0
50
100
SDC
SDC+ACN
SDC
SDC+ACN
Trypsin
GluC
C
Serine
Threonine
Tyrosine
Over-tagging (%)
0
2
4
6
PnTAG:peptide
D
Untagged
NHS-PnTAG
Percent PSMs
0
0.5
1
1.5
2
-136.02
-121.01
-48.99
-18.01
-17.02
-3.14
-2.08
-2.08
-2.06
-2.06
-2.05
-2.01
-1.97
-1.95
-1.95
-1.93
-1.92
-1.90
-0.98
-0.04
0.93
0.96
0.98
1.12
1.89
1.90
2.05
2.05
2.06
2.08
2.94
3.07
17.03
26.01
56.91
57.02
79.96
Mass shift (Da)
