# Supplementary material for: SIMPATICO—Streamlined Isolation of the N-Terminome via Phosphonate Tagging and Coordination-Based Depletion
Source: Mol Cell Proteomics. 2026 Sep 10;25(10):101660. doi: 10.1016/j.mcpro.2026.101660 (PMC13673259; doi:10.1016/j.mcpro.2026.101660)
Supplement: Supplementary file 2 [file mmc2.pdf]

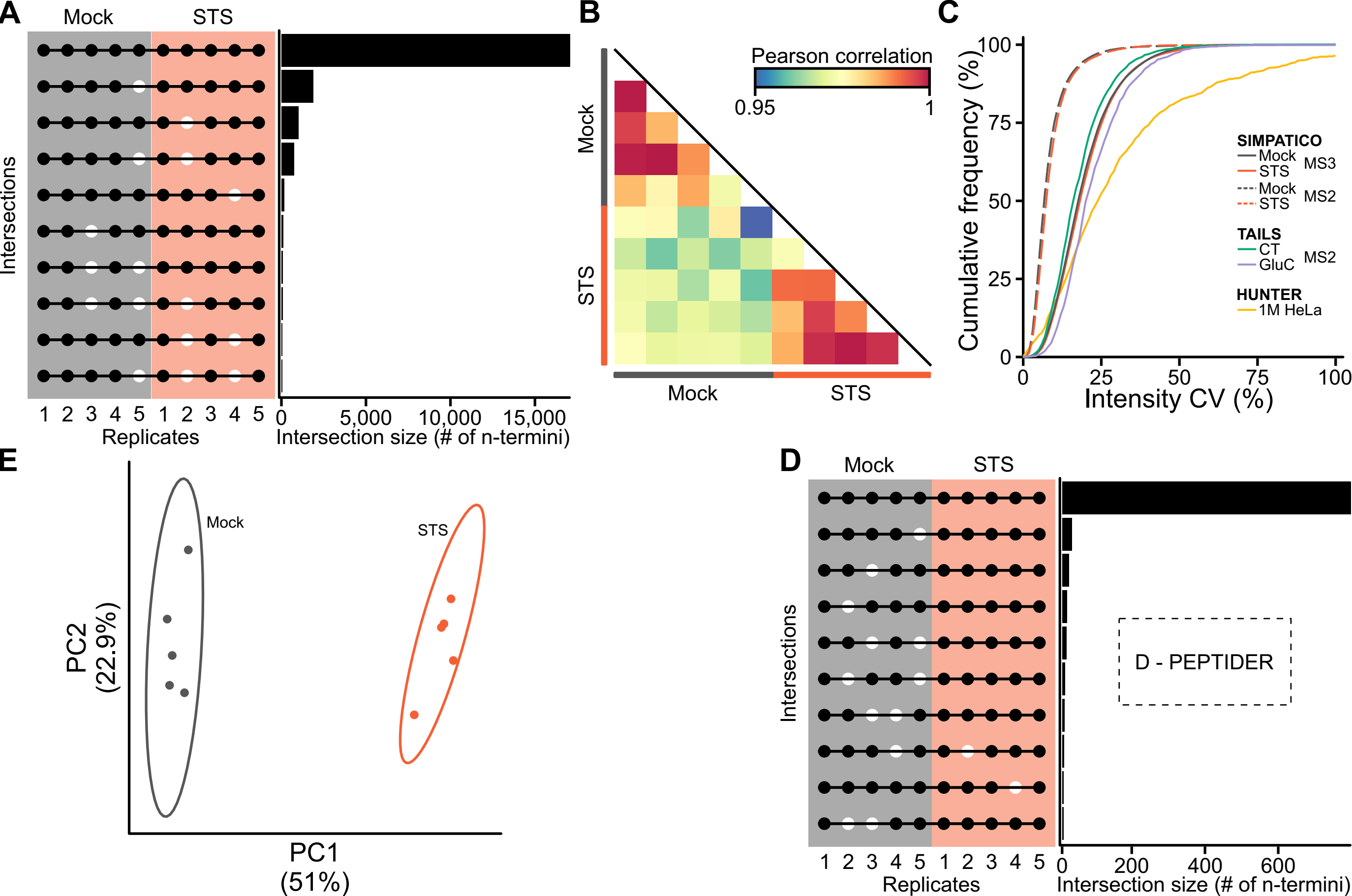

A
Mock
STS
Intersections
1 2 3 4 5 1 2 3 4 5
Replicates
0 5,000 10,000 15,000
Intersection size (# of n-termini)
B
Pearson correlation
0.95
1
Mock
STS
Mock
STS
C
Cumulative frequency (%)
0 25 50 75 100
Intensity CV (%)
SIMPATICO
Mock
STS
MS3
Mock
STS
MS2
TAILS
CT
GluC
MS2
HUNTER
1M HeLa
E
Mock
STS
PC2 (22.9%)
PC1 (51%)
D
Mock
STS
Intersections
1 2 3 4 5 1 2 3 4 5
Replicates
0 200 400 600
Intersection size (# of n-termini)
D - PEPTIDER
