# Supplementary material for: SIMPATICO—Streamlined Isolation of the N-Terminome via Phosphonate Tagging and Coordination-Based Depletion
Source: Mol Cell Proteomics. 2026 Sep 10;25(10):101660. doi: 10.1016/j.mcpro.2026.101660 (PMC13673259; doi:10.1016/j.mcpro.2026.101660)
Supplement: Supplementary file 3 [file mmc3.pdf]

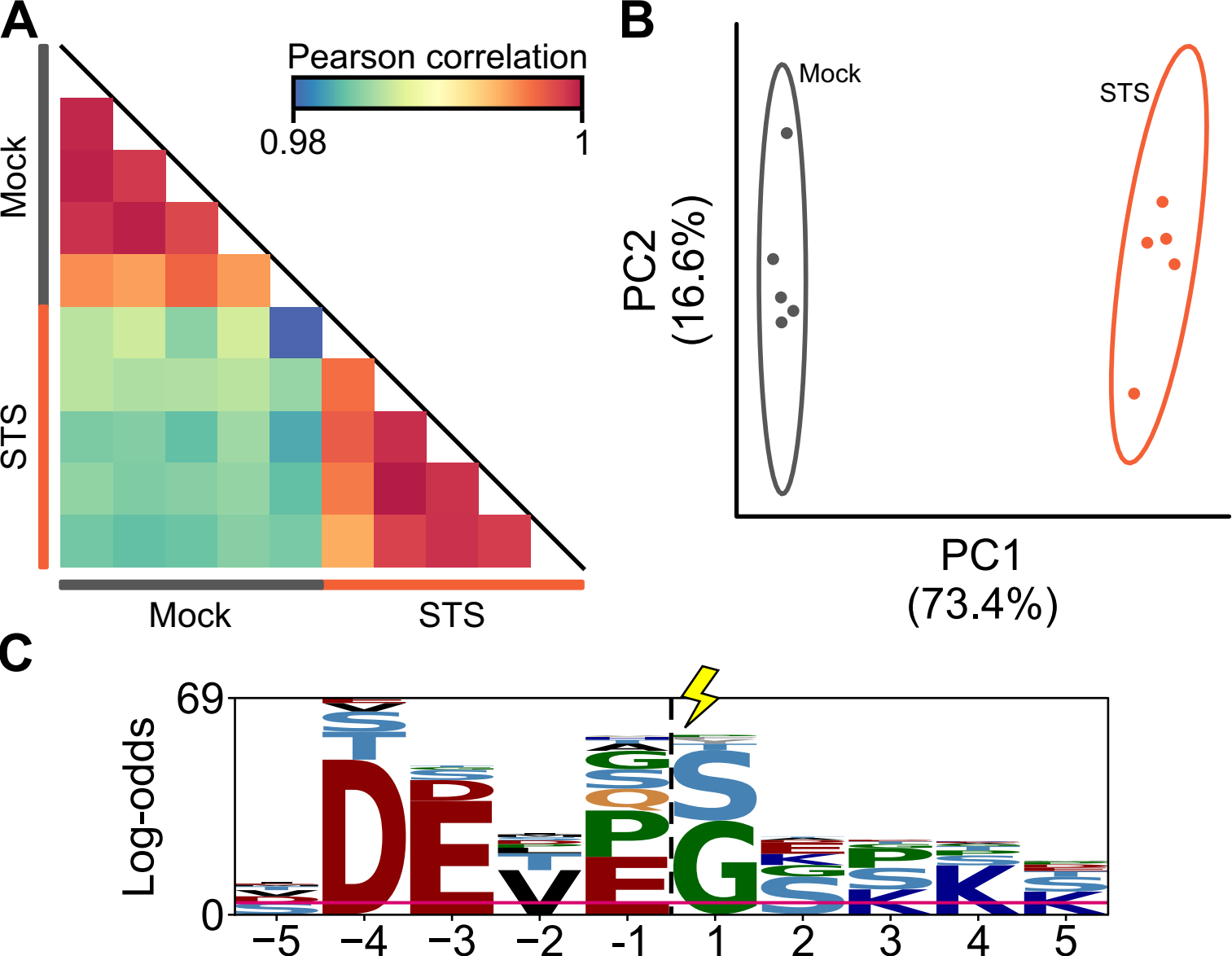
A
Pearson correlation
0.98
1
Mock
STS
Mock
STS
B
Mock
STS
PC2
(16.6%)
PC1
(73.4%)
C
69
0
Log-odds
−5
−4
−3
−2
-1
1
2
3
4
5
