# Supplementary material for: SIMPATICO—Streamlined Isolation of the N-Terminome via Phosphonate Tagging and Coordination-Based Depletion
Source: Mol Cell Proteomics. 2026 Sep 10;25(10):101660. doi: 10.1016/j.mcpro.2026.101660 (PMC13673259; doi:10.1016/j.mcpro.2026.101660)
Supplement: Supplementary file 4 [file mmc4.pdf]

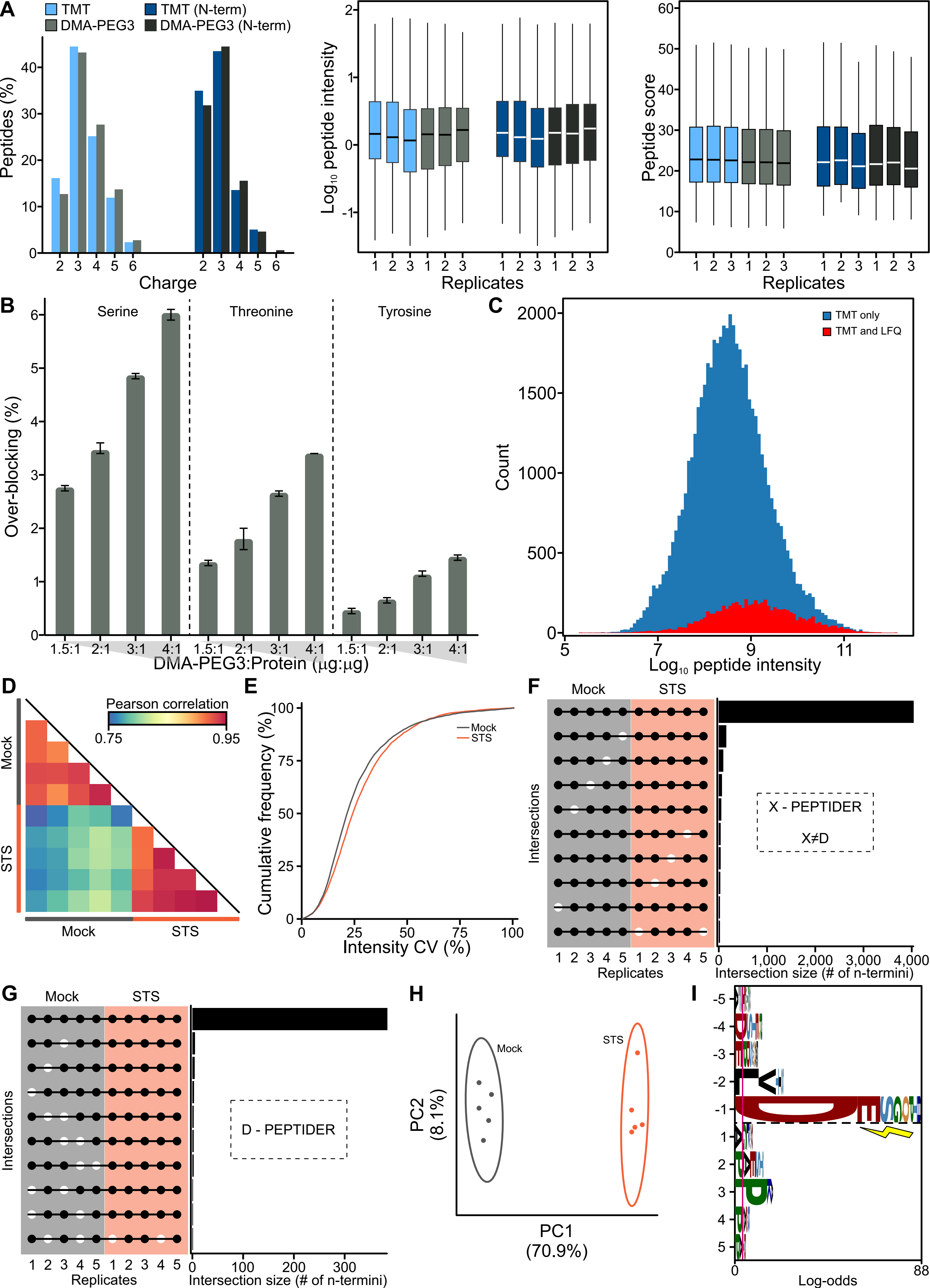

A
TMT
DMA-PEG3
TMT (N-term)
DMA-PEG3 (N-term)
Peptides (%)
Charge
Log10 peptide intensity
Replicates
Peptide score
Replicates
B
Serine
Threonine
Tyrosine
Over-blocking (%)
1.5:1
2:1
3:1
4:1
DMA-PEG3:Protein (µg:µg)
C
TMT only
TMT and LFQ
Count
Log10 peptide intensity
D
Pearson correlation
0.75
0.95
Mock
STS
E
Cumulative frequency (%)
Mock
STS
Intensity CV (%)
F
Mock
STS
Intersections
X - PEPTIDER
X≠D
Replicates
Intersection size (# of n-termini)
G
Mock
STS
Intersections
D - PEPTIDER
Replicates
Intersection size (# of n-termini)
H
Mock
STS
PC2 (8.1%)
PC1 (70.9%)
I
Log-odds
