# Supplementary material for: SIMPATICO—Streamlined Isolation of the N-Terminome via Phosphonate Tagging and Coordination-Based Depletion
Source: Mol Cell Proteomics. 2026 Sep 10;25(10):101660. doi: 10.1016/j.mcpro.2026.101660 (PMC13673259; doi:10.1016/j.mcpro.2026.101660)
Supplement: Supplementary file 5 [file mmc11.docx]

SUPPLEMENTARY INFORMATION

**Supplementary Figure S1.** Evaluation of TMT over-labeling, phosphonate over-tagging, and digestion efficiency of alternative proteases. (A) Bar plot of the percentage of peptide intensity attributable to over-labeled, O-acylated peptides by ester type from the experiment displayed in Figure 1A. For all plots, bars are drawn at the mean value (n = 3), dots indicate individual replicates, and error bars indicate the minimum and maximum values. (B) Bar plots showing the total number of identified peptides (left) and the proportion of peptides containing 0, 1, or ≥2 missed cleavages (right) after digestion with either trypsin or GluC, demonstrating comparable digestion efficiency for both proteases. (C) Bar plot of the percentage of peptide intensity attributable to over-tagged peptides from the analysis displayed in Figure 1B. (D) Distribution of the top 15 modifications within each sample identified by MSFragger via the open search strategy and summarized with PTM-Shepherd, with and without phosphonate tagging. Identifications corresponding to unmodified peptides, isotopic mass shifts, or the PnTAG mass shifts were removed from the analysis. For all plots, bars are drawn at the mean or median value (n = 3), dots indicate individual replicates, and error bars indicate the minimum and maximum values.

**Supplementary Figure S2.** Evaluation of SIMPATICO performance and data reproducibility. (A) Upset plot showing overlap of quantified N-terminal peptides across all samples. (B) Quantitative reproducibility of n-terminal peptide intensities across replicates and experimental conditions. The heatmap depicts Pearson correlation coefficients between samples. (C) Cumulative density plot showing the reproducibility of N-terminal peptide quantification, and comparison with published TAILS (Kalogeropoulos *et al.*) and HUNTER (Weng *et al.*) datasets. Only peptides with a blocked N-terminus (with TMT for SIMAPTICO, TMTpro for TAILS, and dimethyl for HUNTER) were used for the analysis. (D) PCA representation of replicates across experimental conditions. Ellipses are drawn at 95% confidence interval. (E) Same as in (A) but restricted to peptides derived exclusively from aspartic acid cleavage. Only the top 10 intersections are shown for both upset plots.

**Supplementary Figure S3.** Evaluation of SIMPATICO performance from the MS2-based acquisition. (A) Pearson correlation heatmap across five replicates and two conditions. (B) PCA of all samples. (C) Zoom-in of the pLogo plot shown in Figure 2D (MS3 and MS2 combined data).

**Supplementary Figure S4.** Evaluation of LFQ-SIMPATICO performance. (A) Peptide charge state (left), intensity (center), and score (right) distributions for peptides blocked with DMA-PEG3 or TMT in titration experiments performed at a 4:1 reagent-to-protein ratio in 1% SDC (50 μL reaction volume). Distributions are shown for all identified blocked peptides and for the subset of peptides carrying a blocked protein N-terminus. Peptide intensities were normalized to the median intensity of unblocked peptides in each run to correct for minor differences in injected peptide amounts. Variable box widths indicate group sample size. Boxes show the interquartile range (IQR) with the median indicated by a horizontal line. Whiskers extend to ±1.5 × IQR; outliers are not shown. (B) Bar plot of the percentage of peptide intensity attributable to over-blocked peptides across different amounts of DMA-PEG3-NHS reagent, from the analysis displayed in Figure 3B. Bars are drawn at the mean value (n = 3); error bars indicate the minimum and maximum values. (C) Histogram of log N-terminal peptide quantified in the TMT-SIMPATICO experiment from the MS2-based quantification. Red bars indicate N-terminal peptides that were also detected in the LFQ experiment. These peptides are predominantly found at higher intensity values within the distribution. (D) Pearson correlation heatmap across five replicates and two conditions. (E) Cumulative density plot showing the reproducibility of N-terminal peptide quantification. (F) Upset plot showing overlap of quantified N-terminal peptides across all samples. (G) Same as in (F) but restricted to peptides derived exclusively from aspartic acid cleavage. (H) PCA of all samples. (I) pLogo plot of the enriched cleavages (> 2-fold STS-vs-Mock, FDR < 5%) in response to the STS treatment from the LFQ experiment.

**Supplementary Table S1.** TMT protein titration and digestion efficiency experiments.

**Supplementary Table S2.** NHS-PnTAG peptide titration experiments.

**Supplementary Table S3.** N-terminal peptides identification by TMT-SIMPATICO with an MS3-based quantification method.

**Supplementary Table S4.** N-terminal peptides identification by TMT-SIMPATICO with an MS2-based quantification method.

**Supplementary Table S5.** DMA-PEG3-NHS protein titration experiments.

**Supplementary Table S6.** N-terminal peptides identification by LFQ-SIMPATICO.
